# An Uncommon Side Effect of Baclofen Withdrawal in End Stage Renal Disease

**DOI:** 10.7759/cureus.60411

**Published:** 2024-05-16

**Authors:** Krishna Sheth, Dana Odukoya, Kevin Joseph, Aditya Agarwal

**Affiliations:** 1 Internal Medicine, Garnet Health Medical Center, Middletown, USA; 2 Internal Medicine, Touro College of Osteopathic Medicine, Middletown, USA

**Keywords:** altered mental status evaluation, acute metabolic encephalopathy, gaba agonist, baclofen withdrawal, baclofen

## Abstract

Baclofen is a commonly prescribed muscle relaxant that functions to reduce spasticity associated with a variety of musculoskeletal and neurological conditions. Patients with decreased renal function, however, are at an increased risk of suffering from baclofen withdrawal symptoms. We discuss the case of a 77-year-old male who presented to the emergency room with altered mental status and was admitted for acute metabolic encephalopathy found to be from abrupt cessation of baclofen. The presence of kidney disease in this patient further increased his susceptibility to the withdrawal symptoms that began after cessation of the normal medication regimen. This case further illustrates the importance of both comprehensive history and a high index of suspicion when it comes to patient presentation of baclofen withdrawal.

## Introduction

Baclofen is a beta-[4-chlorophenyl]-GABA agonist, commonly referred to as GABA-B agonist. It acts on presynaptic and post-synaptic receptors in the central and peripheral nervous system [1]. Baclofen functions by inhibiting both monosynaptic and polysynaptic reflexes within the spinal cord, allowing for relaxation and decreased spasticity [2]. When baclofen inhibits GABA-B receptors, an influx of potassium ions and efflux of calcium ions cause an overall reduction in the rate of achieving action potential threshold. This mechanism also reduces the amplitude of excitatory postsynaptic potentials that innervate muscle spindles, allowing for the drug to function as a muscle relaxant and reduce spasticity.

Normally, baclofen is absorbed in the gastrointestinal tract following oral administration with peak plasma concentrations seen 2 to 3 hours after ingestion [1]. Baclofen has two main mechanisms of clearance from the body. Roughly 70% is eliminated by renal excretion and 30 % is eliminated via feces [1]. In patients with renal impairment, clearance of baclofen is significantly impaired, increasing its concentration in the body. When abrupt cessation of high levels of the drug occurs, it predisposes the patient to have withdrawal symptoms. Symptoms of baclofen withdrawal syndrome include altered mental status, increased spasticity, fever, nausea, restlessness, seizures, and hallucinations [3]. Recovery from baclofen toxicity can be accelerated by decreasing drug concentration either via cessation of the medication or clearance by hemodialysis [4].

## Case presentation

A 77-year-old male with a past medical history of hypertension, restless leg syndrome, recurrent urinary tract infections, T-cell lymphoma in remission, chronic lower back pain, Crohn’s disease, stage 3 chronic kidney disease (CKD), mitral valve regurgitation, and peripheral vascular disease presented to the emergency room for confusion, minimal responsiveness, and unusual behavior such as hitting himself repeatedly at home. On physical examination, the patient displayed fixed hand wringing and was unable to be redirected. He had been admitted to the hospital four months ago for acute metabolic encephalopathy secondary to left lower extremity cellulitis. During that admission, the patient was administered vancomycin and ceftriaxone with marked improvement. He was discharged on amoxicillin/clavulanic acid and cefalexin for a 14-day total course. The patient’s home medications included acetaminophen 500 mg, baclofen 20 mg, calcitriol 0.25 mcg, calcium carbonate 500 mg, cholestyramine 4g packet, fluoxetine 10 mg, hydralazine 50 mg, potassium chloride 10 meq, ropinirole 2 mg, torsemide 10 mg, tramadol 50 mg, vitamin B12 100 mcg, and vitamin D 1000 units, as seen in Table 1.

**Table 1:** Patient’s home medications, dosage, and frequencies of medications.

Home medications		
Medication	Dosage	Frequency
Acetaminophen	500 mg	Every six hours as needed
Baclofen	20 mg	Three times a day
Calcitriol	0.25 mcg	One tablet a day
Calcium carbonate	500 mg	Two tablets a day
Cholestyramine	4g packet	Two times a day
Fluoxetine	10 mg	One table a day
Hydralazine	50 mg	Three times a day
Potassium chloride	10 mEq	Two times a day
Ropinirole	2 mg	Every six hours
Torsemide	10 mg	One tablet a day
Tramadol	50 mg	Four times a day as needed
Vitamin B-12	100 mcg	One tablet a day
Vitamin D	2,000 units	One tablet a day

**Figure 1:**
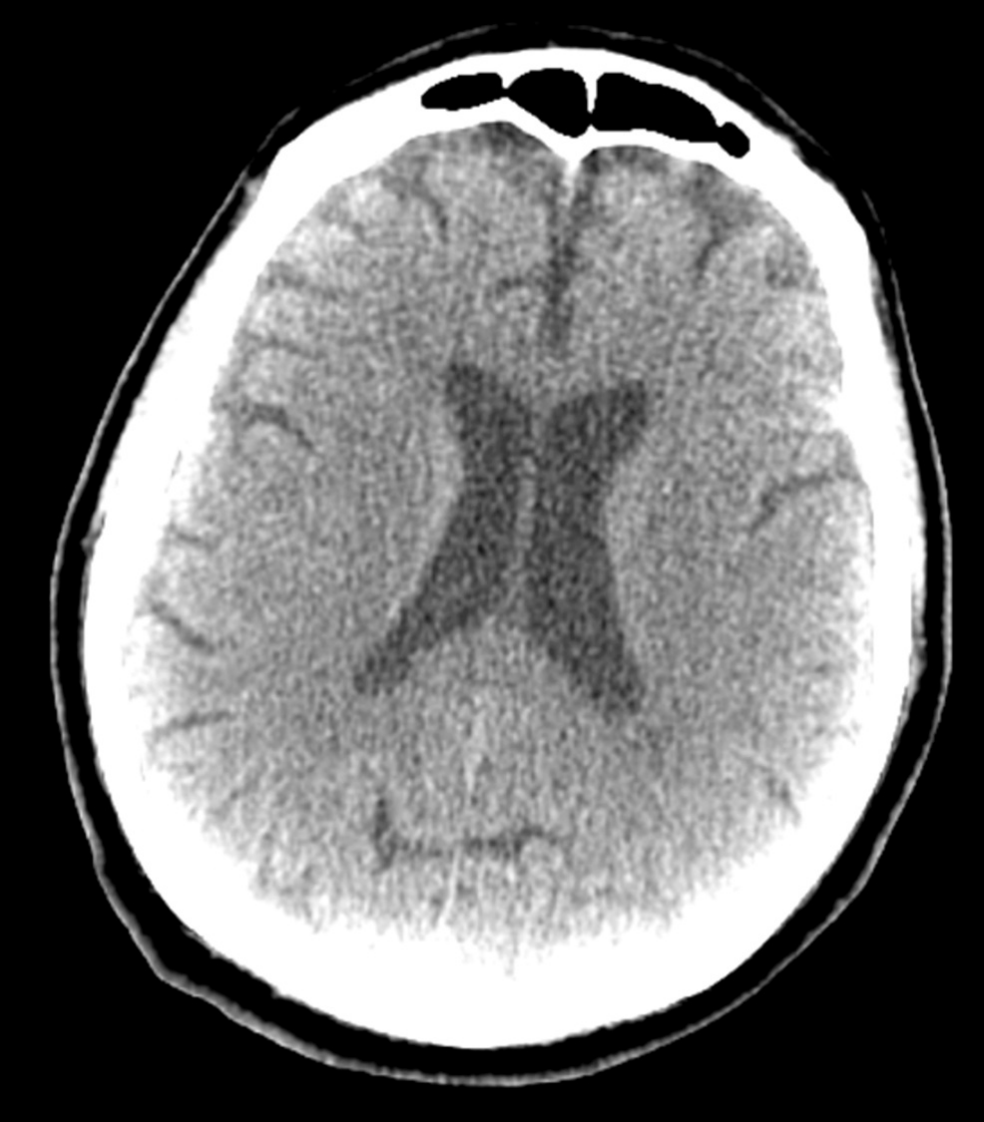
CT brain without contrast shows that the fourth ventricle is midline, and ventricles and subarachnoid space are normal in size. There is no evidence of acute infarct, edema, or hemorrhage.

In the emergency department (ED), vital signs were stable. The basic metabolic panel showed a creatinine level of 3.00 mg/dL and blood urea nitrogen of 69 mg/dL, illustrating acute kidney injury with stage 3 CKD. The patient also presented with non-anion gap metabolic acidosis demonstrated by CO2 level of 13 mmol/L which had significantly decreased from 23 mmol/L on previous admission. Chest x-ray, CT brain, and ultrasound venous doppler were all negative. CT chest, abdomen, and pelvis displayed minimal bilateral dependent atelectasis, moderate hiatal hernia, and a moderate amount of stool. CT brain without contrast showed the fourth ventricle midline, and ventricles and subarachnoid space normal in size. There was no evidence of acute infarct, edema, or hemorrhage, as shown in Figure 1. The patient was administered vancomycin, piperacillin/tazobactam, midazolam, and 1-liter normal saline bolus in the ED and was admitted for acute metabolic encephalopathy. 

**Figure 2:**
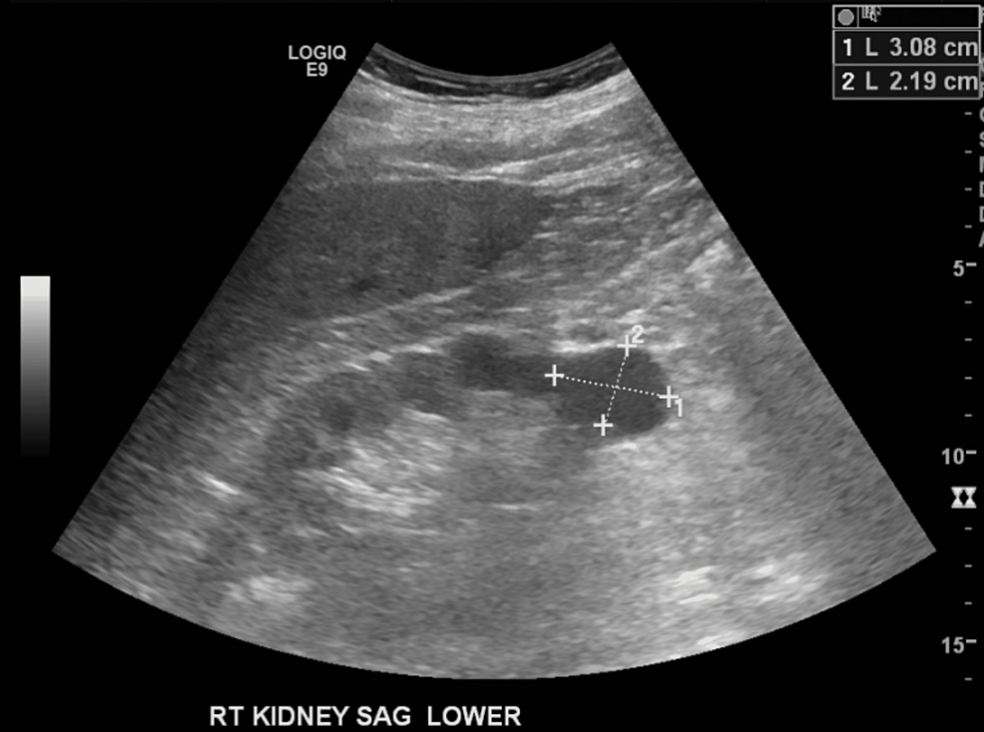
Renal ultrasound shows a simple cyst in the lower pole measuring 3.1 x 2.2 x2.3cm shown by 1 and 2. No evidence of mass, calcification, or hydronephrosis was noted.

The patient was admitted to the hospital with stable vital signs but was oriented only to the person. Given elevated creatinine at 3.0 mg/dL, nephrology recommended a renal ultrasound, which showed a simple cyst; however, there was no acute pathology, as seen in Figure 2.

**Figure 3:**
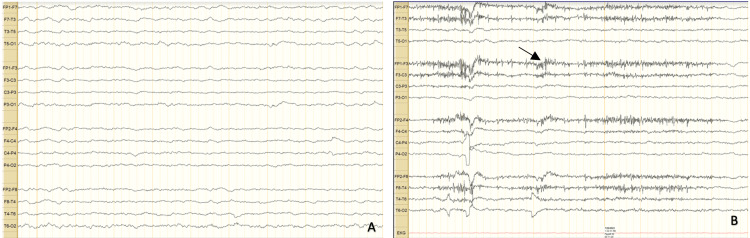
Electroencephalogram (EEG) results. (A) The baseline diffuse slow wave slowing while the patient is asleep. (B) Baseline diffuse slowing while the patient is awake. Eye blink and artifacts, seen by an arrow, are noted over frontal regions. The finding of mild diffuse slowing noted throughout the study suggests some degree of underlying diffuse cerebral dysfunction which is non-specific etiology. However, there was no evidence of electrical seizure activity or epileptogenic features.

Per neurology consult, an electroencephalogram (EEG) was obtained and displayed diffuse slowing with diffuse cerebrum dysfunction. No electrical seizure activity or epileptogenic features were evident, as seen below in Figure 3. Upon thorough history taking it was discovered that he had stopped taking his baclofen for an unknown number of days prior to admission. Following this disclosure, the medical team decided to reinstate his baclofen regimen of 20 mg every six hours. The patient showed marked improvement in a few days and was discharged shortly after and advised to continue taking the baclofen 20 mg as prescribed. 

## Discussion

Baclofen withdrawal may occur in patients who are administered oral baclofen. Withdrawal symptoms typically manifest within hours to days following cessation of medication [2]. Symptoms are non-specific and may span various ailments; thus a high index of suspicion is required in the clinical diagnosis of withdrawal. Amongst the typical symptoms, a patient may present with altered mental status, spasticity exacerbation, fever, nausea, weakness, and autonomic instability, as seen in our patient [2]. 

The patient presented above had a history of stage 3 CKD and presented with an acute kidney injury as evidenced by his elevated creatinine and blood urea nitrogen level on labs. The patient had also previously stopped taking his baclofen as prescribed for his lower back pain a few days prior. Decreased kidney function contributes to lower drug elimination. This buildup of the drug allows the body to be exposed to increased levels of drug concentration. Abrupt cessation of the drug allows the body to be more predisposed to various withdrawal symptoms, including central nervous system (CNS) involvement.

Treatment of baclofen withdrawal includes frequent monitoring of vitals, prevention of dehydration, pulmonary support, cardiac support, and the resumption of baclofen dosing via the original route of administration [2]. Rhabdomyolysis represents one of the more severe potential side effects of withdrawal. This is oftentimes prevented through intravenous fluids. Since baclofen is mainly excreted through the kidneys without being altered, baclofen withdrawal is more commonly seen in patients with end stage renal disease (ESRD) [5]. Per literature review, patients with ESRD have a 68% chance of developing baclofen toxicity and thus baclofen withdrawal [6]. Thus far, roughly 41 cases of baclofen toxicity have been reported in CKD patients however these patients are mostly dialysis-dependent [7]. A review of 41 reported cases of baclofen toxicity illustrates that elderly and dialysis-dependent patients are more likely to develop toxicity [7]. Withdrawal symptoms such as acute metabolic encephalopathy manifest because the drug can effectively cross the blood-brain barrier allowing for CNS depression and altered mental status [8].

## Conclusions

Patients with decreased renal function are at an increased risk of suffering from baclofen toxicity and baclofen withdrawal upon abrupt cessation of the drug. Baclofen withdrawal may present with symptoms such as altered mental status, increased spasticity, fever, nausea, and autonomic instability. Due to typical symptoms that are frequently seen with other etiologies, physicians must have a high index of suspicion for accurate diagnosis. It is also essential that physicians rule out other possible causes for the symptoms that are typically seen with baclofen withdrawal. In the patient presented above, quick resolution of symptoms was seen with reinstatement of the prescribed dose of baclofen. Oftentimes, patients will exhibit a significant reduction of symptoms through administration of oral baclofen at the same dosage as previously prescribed. 
